# Manufacturing of Microfluidic Devices with Interchangeable Commercial Fiber Optic Sensors

**DOI:** 10.3390/s21227493

**Published:** 2021-11-11

**Authors:** Krystian L. Wlodarczyk, William N. MacPherson, Duncan P. Hand, M. Mercedes Maroto-Valer

**Affiliations:** 1 Research Centre for Carbon Solutions (RCCS), School of Engineering and Physical Sciences, Heriot-Watt University, Edinburgh EH14 4AS, UK; M.Maroto-Valer@hw.ac.uk; 2 Applied Optics and Photonics (AOP) Group, School of Engineering and Physical Sciences, Heriot-Watt University, Edinburgh EH14 4AS, UK; W.N.MacPherson@hw.ac.uk (W.N.M.); D.P.Hand@hw.ac.uk (D.P.H.)

**Keywords:** microfluidic devices, lab-on-a-chip, fiber optic sensors, pH sensors, pressure sensors, laser materials processing, laser manufacturing, microwelding, glass, additive manufacturing

## Abstract

In situ measurements are highly desirable in many microfluidic applications because they enable real-time, local monitoring of physical and chemical parameters, providing valuable insight into microscopic events and processes that occur in microfluidic devices. Unfortunately, the manufacturing of microfluidic devices with integrated sensors can be time-consuming, expensive, and “know-how” demanding. In this article, we describe an easy-to-implement method developed to integrate various “off-the-shelf” fiber optic sensors within microfluidic devices. To demonstrate this, we used commercial pH and pressure sensors (“pH SensorPlugs” and “FOP-MIV”, respectively), which were “reversibly” attached to a glass microfluidic device using custom 3D-printed connectors. The microfluidic device, which serves here as a demonstrator, incorporates a uniform porous structure and was manufactured using a picosecond pulsed laser. The sensors were attached to the inlet and outlet channels of the microfluidic pattern to perform simple experiments, the aim of which was to evaluate the performance of both the connectors and the sensors in a practical microfluidic environment. The bespoke connectors ensured robust and watertight connection, allowing the sensors to be safely disconnected if necessary, without damaging the microfluidic device. The pH SensorPlugs were tested with a pH 7.01 buffer solution. They measured the correct pH values with an accuracy of ±0.05 pH once sufficient contact between the injected fluid and the measuring element (optode) was established. In turn, the FOP-MIV sensors were used to measure local pressure in the inlet and outlet channels during injection and the steady flow of deionized water at different rates. These sensors were calibrated up to 140 mbar and provided pressure measurements with an uncertainty that was less than ±1.5 mbar. Readouts at a rate of 4 Hz allowed us to observe dynamic pressure changes in the device during the displacement of air by water. In the case of steady flow of water, the pressure difference between the two measuring points increased linearly with increasing flow rate, complying with Darcy’s law for incompressible fluids. These data can be used to determine the permeability of the porous structure within the device.

## 1. Introduction

Precise and contact-free manipulation, high-throughput (parallel) processing, real-time analysis, and visual observation of very small amounts of fluids (from picoliters to microliters) are possible thanks to microfluidic devices that play an important role in different branches of life sciences, especially in biomedical and biotechnological applications, as well as in hydrology, petroleum engineering, and carbon dioxide (CO_2_) storage research. Microfluidic devices are widely used e.g., for drug development and delivery, cellular manipulation and analysis, viral detection, tissue engineering, genetic analysis, and rapid diagnostics as low-cost disposable bioassays [1,2,3,4,5,6,7,8,9,10]. In hydrology, microfluidic chips are used to test water quality, including the detection of waterborne pathogens (e.g., *E. coli* bacteria) and inorganic pollutants (e.g., heavy metals) [11]. In petroleum engineering and CO_2_ storage research, they are often used as physical models of porous media to study different phenomena related to flow, transport, and reactive processes on the microscale (pore level), e.g., the displacement of immiscible fluid phases, fluid trapping and saturation, precipitation of minerals, and dissolution of solvents and chemical compounds [12,13,14,15,16,17,18,19,20].

In situ monitoring of parameters such as temperature, pressure, pH, O_2_ concentration, CO_2_ concentration, electrical impedance, and thermal conductivity is highly desirable in many of the aforementioned applications. For instance, local measurement of pH value, O_2_ concentration, and/or CO_2_ concentration in microfluidic devices allows us to monitor and optimize process conditions (e.g., for efficient cell growth [21]), detect various substances (e.g., waterborne contaminants [11]), and observe chemical reactions (e.g., mineral precipitation [18]). In turn, the measurement of pressure at specific locations in a microfluidic device allows us to determine the permeability of the pattern [22] as well as correlate pressure changes with visual observations at the microscale in order to explain processes that govern subsurface systems (e.g., hydrocarbon reservoirs) [23]. Local measurements of thermal conductivity and electrical impedance can also be useful, e.g., to detect cellular response during culture [24], assess blood samples [25], count particles [26], determine the sample position in a microfluidic channel [27], or differentiate fluids in multi-phase fluid flow experiments [28].

When sensors are integrated within a microfluidic device, they increase the functionality and value of the device. Unfortunately, the fabrication of bespoke microfluidic devices with integrated sensors can be difficult, time consuming, and expensive because this requires advanced know-how and specialized equipment e.g., to develop a sensor, perform its accurate deposition and patterning, and seal the microfluidic chip without damaging the sensor [23,24,25,26,27,28,29,30]. Such microfluidic devices may also impose some limitations in conducting experiments. For instance, it may not be possible to reuse the microfluidic device in the same or another experiment because the sterilization processes and solvents used to clean microfluidic patterns can damage or significantly deteriorate the performance of permanently embedded sensors [30]. Under such circumstances, the only solution is to use a new microfluidic device, which increases the cost of the experiment.

In this article, we describe an easy-to-implement method that allows flexible and “reversible” integration of commercially available fiber optic sensors within microfluidic devices. To integrate sensors, we designed custom connectors and manufactured them using a stereolithography 3D printer. The key feature of the presented integration method is that various “off-the-shelf” fiber optic sensors can be connected to the device and safely disconnected if necessary, without damaging the microfluidic chip under test. This is a significant difference to the integration method described by Zarikos et al. [23], who permanently embedded miniature fiber optic pressure sensors in a microfluidic device manufactured from polydimethylsiloxane (PDMS).

Two different fiber optic sensors are used as demonstrators in our work. The first are pH sensor prototypes named “pH SensorPlugs” that were provided for testing by PreSens Precision Sensing GmbH (Germany) [31]. These relatively small sensors (diameter of approximately 2.8 mm) enable a localized measurement of pH; therefore, they can be used in microfluidic devices for real-time monitoring of process conditions (e.g., during cell growth or when mixing different fluids in microreactors) and also for in situ detection of chemical reactions (e.g., precipitation of minerals). The second sensors used in our work are miniature pressure sensors named “FOP-MIV” manufactured by FISO Technologies Inc. (Canada) [32]. These sensors were developed primarily for medical applications [32,33]. However, due to their very small footprint (diameter of only 0.55 mm), they are suitable for use with microfluidic devices, e.g., to measure pressure drop across the porous structure to define the pattern permeability; or to investigate dynamics of fluids in the pore networks in order to better understand the processes governing subsurface systems at the macroscale [23].

## 2. Materials and Methods

### 2.1. Microfluidic Device

**Figure 1:**
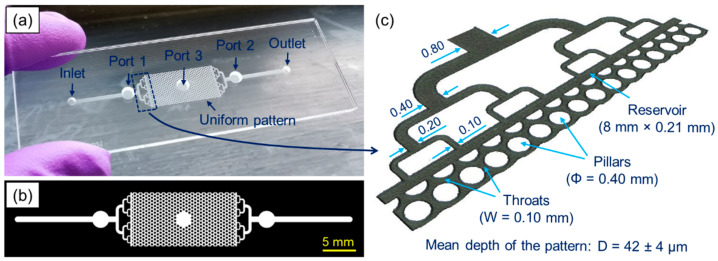
(**a**) Laser-manufactured, glass microfluidic device with three round ports (Ports 1–3) dedicated to attaching commercial fiber optic sensors, (**b**) schematic of the overall microfluidic pattern, and (**c**) dimensions of the key features (given in mm) measured at Full-Width-Half-Maximum using an Alicona G4 InfiniteFocus 3D surface profilometer.

To demonstrate how commercially available fiber optic sensors can be integrated with microfluidic devices, we manufactured a test microfluidic device from two borosilicate glass plates (Borofloat 33, SCHOTT Technical Glass Solutions GmbH, Germany) with dimensions of 75 mm × 25 mm × 1.1 mm (see Figure 1). The device was manufactured using only an ultrashort pulsed picosecond laser (TruMicro 5x50, TRUMPF GmbH + Co. KG, Germany), following the fabrication procedure described in [34]. The laser was used to: (a) drill an inlet, outlet, and three additional through-holes with a diameter of 2.8 mm in a first glass plate, (b) generate a microfluidic pattern on the surface of a second glass plate by ablating the material, and (c) permanently close the microfluidic pattern by placing the first glass plate (with the holes) on the top of the second glass plate (with the pattern), bringing and keeping the plates in very close contact (less than 1 µm apart), and then welding them together. The glass plates are bonded together by focusing the laser beam to a very small spot (diameter < 4 µm) approximately 150 µm below the glass–glass interface, giving rise to non-linear optical effects (i.e., multi-photon and avalanche absorption), which in turn results in plasma formation. The plasma expands upwards, crossing the glass–glass interface and mixing the molten glass that subsequently cools to form a permanent weld when the laser beam moves away. As described in our previous publications [34,35], the entire fabrication process takes only a few hours and allows rapid prototyping and manufacturing of bespoke custom microfluidic devices.

The microfluidic device tested has a uniform pore network pattern (15.58 mm long and 8.00 mm wide) that represents a ‘generic’ porous test structure. Such, and similar, patterns are often used as benchmarks to investigate flow, transport, and reactive processes in porous media [17,18,19,20]. The pattern consists of circular pillars with a diameter of 0.4 mm, which are evenly spaced from each other by 0.5 mm, creating a uniform network of microchannels with 0.1 mm wide throats (see Figure 1c). An average depth of the pattern is 42 ± 4 µm, as measured using a 3D surface profilometer (Alicona G4 InfiniteFocus, Alicona Imaging GmbH, Austria). The pattern does not have pillars in the center (see Figure 1b) because this area was reserved for an additional sensor port. On both sides of the pattern, there are rectangular areas (8.00 mm × 0.21 mm) and bifurcated channels that were designed to evenly distribute the injected fluid into the uniform pattern. The channel dimensions are given in Figure 1c. At a distance of 12.5 mm from the inlet and the outlet (see Figure 1b), there are also two circular areas with a diameter of 2.5 mm (called Port 1 and Port 2), which were reserved to embed the sensors. The volume of the pattern between Ports 1 and 2 was calculated to be 3.1 µL, whereas its total volume is 3.9 µL.

### 2.2. Sensors and Interfacing

The key parameters of the pH SensorPlugs and FOP-MIV sensors are summarized in Table 1.

#### 2.2.1. pH SensorPlugs

The pH SensorPlugs (SPL-ML-HP5) are built upon commercial male mini-Luer connectors onto which a thin, pH-sensitive disc (fluorescent optode) with a diameter of approximately 1.8 mm is attached. As specified by the manufacturer, these sensors allow real-time measurement of pH in the range of 5.5 to 8.5 with an accuracy of ±0.05 pH. The sensors are delivered pre-calibrated, so they are ready for use immediately after connecting to appropriate pH readout units (pH-1 SMA HP5-v2, PreSens Precision Sensing GmbH, Germany). Since the pH may slightly change with temperature, one readout unit was connected to a Pt100 temperature probe (also supplied by PreSens) to measure the ambient temperature near the sensors. The pH readout units were connected to a computer and controlled in the PreSens Measurement Studio 2 RC software. The pH values were measured at a sampling rate of 0.33 Hz.

#### 2.2.2. FOP-MIV Pressure Sensors

**Figure 2:**
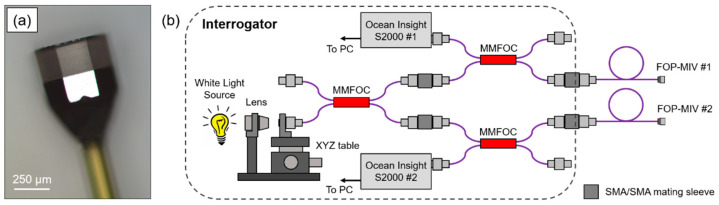
(**a**) Optical microscope image of the FOP-MIV sensor and (**b**) schematic of the in-house designed, bespoke interrogation system used to acquire changes in the FP cavity length of the FOP-MIV sensors.

The principle component of the FOP-MIV sensors (see Figure 2a) is a Fabry–Pérot (FP) cavity attached to the end of an optical fiber [32]. The FP cavity is made of two parallel reflecting surfaces. One of them is a thin diaphragm that deflects under the influence of external pressure. When the diaphragm deflects, the length of the FP cavity alters, and this can be detected using an optical signal interrogation system suitable for the measurement of the Free Spectral Range (FSR) of the FP cavity. The FSR is a measure of the separation of reflection peaks in the optical frequency domain (1/wavelength). As the external pressure exerted on the sensor increases, the length of the FP cavity becomes shorter and the distance between the peaks (and hence the FSR) increases.

Figure 2b shows a schematic of the in-house designed, bespoke interrogation system that was used to measure changes in the FP cavity length of the FOP-MIV sensors. The interrogation system comprises a tungsten bulb (white light source), a focusing lens (×10 0.25 NA microscope objective), three 2 × 2 multi-mode fiber optic couplers (MMFOC) with a 50/50 split of optical power (TM50R5S2A, Thorlabs Inc., USA), four SMA to SMA mating sleeves, and two spectrometers (S2000, Ocean Insight, USA). The control of the S2000 spectrometers, data acquisition, and real-time processing of the optical spectra from both sensors were performed in LabVIEW (National Instruments, USA) using a bespoke interrogation algorithm.

### 2.3. Custom Connectors

**Figure 3:**
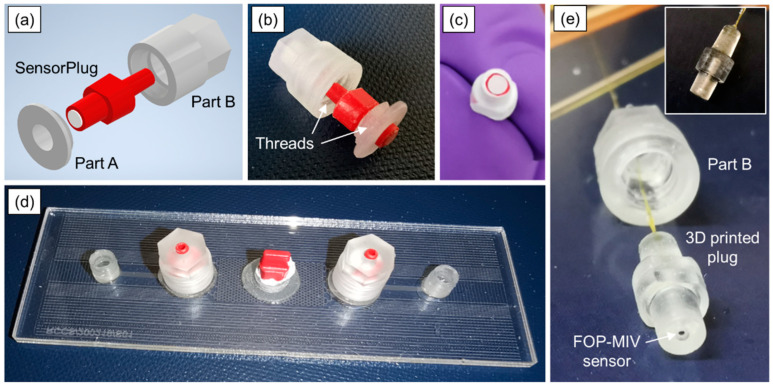
Three-dimensional (3D)-printed connectors used to embed the pH SensorPlugs and FOP-MIV sensors into the ports of the glass microfluidic devices: (**a**) 3D design and (**b**) printed Parts A and B with a fitted pH SensorPlug, (**c**) pH SensorPlug with PTFE tape, (**d**) microfluidic device with all components attached (including the pH SensorPlugs), and (**e**) 3D printed plug with a built-in FOP-MIV sensor.

The pH SensorPlugs and FOP-MIV sensors were attached to the laser-manufactured glass microfluidic device by means of custom connectors that were designed and manufactured from clear photocurable resin (FLGPCL04, Formlabs Inc., Somerville, MA, USA) using a desktop, stereolithography 3D printer (Form 2, Formlabs, USA). The custom connectors consist of two parts, as illustrated in Figure 3a. Part A has a short external thread, while Part B has an internal thread (see Figure 3b). Parts A were attached to all three ports of the microfluidic device using epoxy adhesive (Araldite Standard, Huntsman Advanced Materials BVBA, Belgium), whilst Parts B securely held the sensors in the ports after screwing them to Parts A, as shown in Figure 3d. 

To ensure tight connections, the pH SensorPlugs were carefully wrapped with PTFE tape (see Figure 3c) before being inserted into the ports. Since only two pH readout units were available, simultaneous pH measurement could only be performed at two locations. Therefore, the pH SensorPlugs were connected to Ports 1 and 2 (see Figure 1a), so that the pH values could be monitored in the inlet and outlet channels close to the uniform pattern, while Port 3 was sealed using a commercial male mini-Luer plug (Fluidic 334, Microfluidic ChipShop GmbH, Jena, Germany). The inlet and outlet had the so-called stand-alone female mini-Luers (Fluidic 630, Microfluidic ChipShop GmbH, Jena, Germany) that were attached to the glass surface with the Araldite Standard glue.

The same microfluidic device was used to test the FOP-MIV pressure sensors. Since Parts A were permanently attached to the device, the FOP-MIV sensors required special adapters that would allow the sensors to be connected using the existing Parts A and B. For this purpose, additional custom male mini-Luer plugs (see Figure 3e) were manufactured from clear photocurable resin using the Formlabs 3D printer. The adapters have a through hole with three different diameters (see the insert of Figure 3e). The first section of the hole has a length of 1 mm and a diameter of 0.8 mm, so it is large enough to accommodate and protect the sensor from damage while handling and inserting the plug into Part A. The middle section of the hole has a diameter of only 0.3 mm, and hence, it is smaller than the sensor body but still is larger than the diameter of the optical fiber. This section was designed to prevent the sensor from being inserted too far into the plug during its assembly. The third section of the hole has a diameter of 1.0 mm and is large enough to apply a small amount of glue (Araldite Standard) to fix the optical fiber and seal the plug from the top.

### 2.4. Recalibration and Testing of pH SensorPlugs

**Figure 4:**
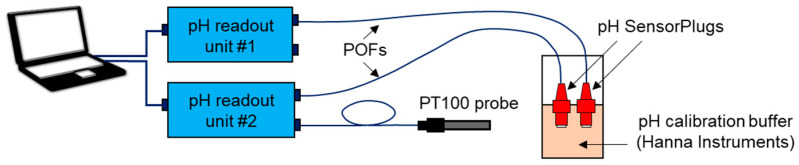
Setup used for the single point calibration of PreSens pH SensorPlugs.

As recommended by the manufacturer, the pH SensorPlugs were recalibrated before they were attached to the microfluidic device. Recalibration was performed using pH 7.01 calibration buffer (HI-70007P, Hanna Instruments Ltd., Leighton Buzzard, UK) that has a nominal pH value of 7.03 at 20 °C, which is within the sensor measurement range. The sensors were immersed in 20 mL of the buffer for at least 15 min (see Figure 4), and then, single-point calibration was performed in PreSens Measurement Studio 2 RC.

Following recalibration, the pH SensorPlugs were inserted into Ports 1 and 2 and secured with Part B, as shown in Figure 3d. Then, the sensors were connected to the pH readout units by means of polymer optical fibers (POFs). The inlet of the microfluidic device was connected to a 1 mL plastic Luer-lock syringe using a PTFE tube and a male mini-Luer connector (Fluidic 331, Microfluidic ChipShop GmbH, Jena, Germany). The syringe was filled with pH 7.01 buffer solution. Any air bubbles in the syringe and PTFE tube were purged before the tubing was connected to the inlet of the microfluidic device. The outlet was open to atmosphere, and the exiting fluid was collected in a vial. The pH buffer was injected into the microfluidic pattern with a constant rate using a syringe pump (AL-300, World Precision Instruments, Sarasota, FL, USA) that ensured a dispensing accuracy of ±1%. The setup also included a 5 Megapixel USB camera, which allowed us to observe the flow of the pH buffer through the microfluidic pattern. The pH values were recorded with a frequency of 0.33 Hz (i.e., every 3 s). The integrity of the ports was regularly inspected to detect possible leaks.

### 2.5. Calibration and Testing of FOP-MIV Sensors

The FOP-MIV sensors are usually delivered with an electronic chip attached to the fiber optic connector. The chip contains calibration data that can be read with an appropriate reading unit, e.g., the SKR module manufactured by FISO Technologies Inc. [32]. However, in this work, the SKR reading unit was unavailable, and therefore, it was necessary to calibrate the sensors before they were attached to the microfluidic device. To do so, the FOP-MIV sensors were calibrated against hydrostatic pressure (*P_h_*), immersing the sensors at different depths (D) in a 145 cm high glass cylinder filled with water. The *P_h_* values were calculated using the following equation [36] (p. 263):

(1) $$P_{h}=\rho \cdot g\cdot D$$

where ρ is the density of water (997 kg/m^3^) and g is the standard acceleration due to gravity (9.81 m/s^2^). The glass column allowed us to obtain hydrostatic pressures in the range of 0 to 136.9 mbar with an increment of 0.978 mbar/cm. 

**Figure 5:**
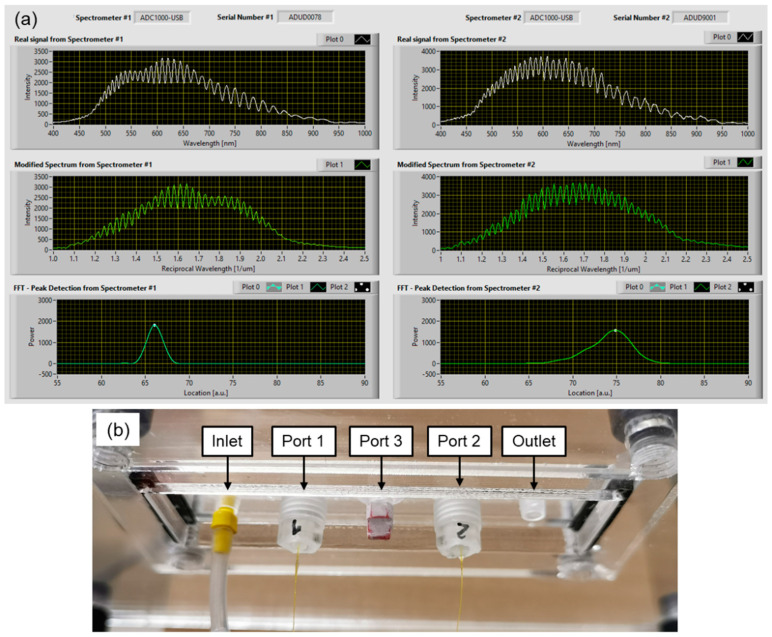
(**a**) Reflection spectra acquired by the spectrometers when the FOP-MIV sensors are connected to the interrogation system, and (**b**) close-up on the FOP-MIV sensors attached to the microfluidic device via Port 1 and Port 2.

The interrogation system shown in Figure 2b allowed us to measure the FSR from the reflected spectrum of the Fabry–Pérot cavity (see Figure 5a). When the spectrum is plotted as a function of reciprocal wavelength, the distance between reflection peaks becomes constant, and this characteristic frequency is a measure of the FSR. This is recovered in the Fourier domain, where the dominant frequency is represented by a characteristic peak whose position can be monitored. Any increase of the external pressure exerted on the sensor causes a decrease of the length of the FP cavity, and this shifts the characteristic peak toward zero.

Following the calibration process, the 3D-printed plugs with built-in FOP-MIV sensors were carefully wrapped with PTFE tape, as shown in Figure 3c. Then, they were inserted into Ports 1 and 2 and secured with Part B, as shown in Figure 5b. The middle port (Port 3) was sealed with a male mini-Luer plug, and the outlet was open to atmosphere, while the inlet was connected to a plastic Luer-lock syringe using a PTFE tube. This time, the syringe was filled with deionized (DI) water. Before connecting the syringe to the inlet of the microfluidic device, air bubbles were purged from the syringe and PTFE tube. Pressures in Ports 1 and 2 were recorded with a frequency of 4 Hz.

At the beginning, when the microfluidic pattern was filled with air, DI water was injected with a rate of 4 µL/min. The displacement of air by water was recorded with the USB camera, so that later, some pressure changes in the measured sites could be explained. When the water saturated the pattern, the flow rate was changed, and the pressures in Ports 1 and 2 were recorded for at least 1 min. Such measurements were made for several different flow rates (from 2 to 20 µL/min). 

## 3. Results and Discussion

### 3.1. pH SensorPlugs

Following the single-point calibration, the pH SensorPlugs were inserted to Ports 1 and 2 and secured with the 3D-printed parts (Parts B). Then, the pH calibration buffer was injected into the pattern with a rate of 2 µL/min. The buffer filled was observed to almost fill the entire pattern in approximately 2 min. After this time, the fluid distribution in the pattern remained unchanged until the end of the experiment. 

**Figure 6:**
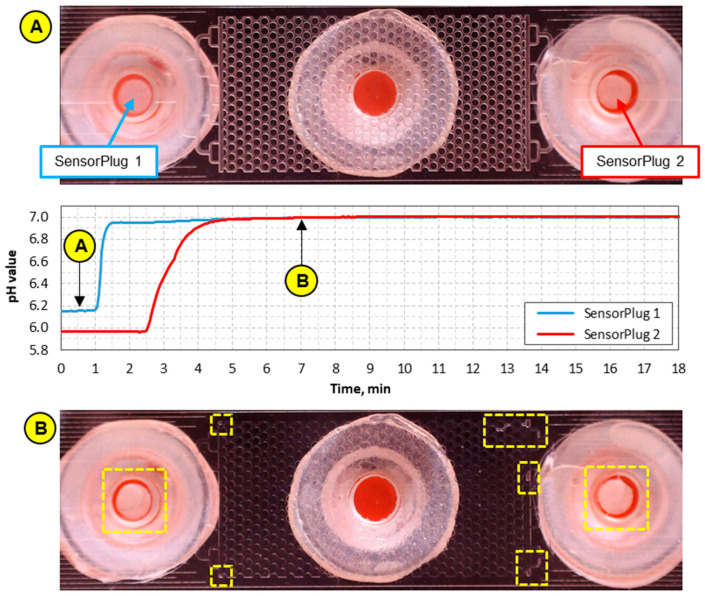
pH values recorded by the SensorPlugs during the injection and flow of pH 7.03 buffer with a rate of 2 µm/min. Images (**A**) and (**B**) were taken before and during the injection of the buffer, respectively. The boxes in Image (**B**) indicate the areas with trapped air.

Figure 6 shows pH values measured by the pH SensorPlugs during the injection of pH 7.03 calibration buffer with a rate of 2 µL/min. Initially, when the microfluidic pattern was filled with air, the pH values measured by SensorPlug 1 and SensorPlug 2 were invalid (pH 6.15 ± 0.01 and pH 5.96 ± 0.01, respectively) because these sensors are suitable only for liquids with a minimum ionic strength of about 50 mM and a minimum buffer capacity of about 2 mM. However, when the pH buffer arrived at Port 1, as observed with the camera, SensorPlug 1 responded to the fluid and the pH value started to increase. The signal rise time was 24 s (here defined for a 90% increase from an initial value to a final value), whilst a final pH value of 7.00 was obtained after a few minutes. This pH value is only 0.03 lower than that expected and is within the sensor accuracy range. The slightly lower values may result from the so-called “bleaching” because the SensorPlugs are considered disposable components and are expected to start losing the original accuracy after a certain number of illuminations. SensorPlug 2 detected a change in pH with a 90 s delay with respect to SensorPlug 1. This delay, as confirmed by the camera, is in agreement with our expectations because the volume of the pattern between Ports 1 and 2 is approximately 3 µL and hence is expected for fluid to take this time to fill the pattern based upon the injection rate of 2 µL/min. As shown in Figure 6, SensorPlug 2 responded differently from SensorPlug 1 because the signal rise time is 90 s (not 24 s). Nevertheless, after this time, both sensors recorded the same pH value. 

The images captured by the USB camera allowed us to observe small air bubbles that were trapped not only in the pattern but also in Ports 1 and 2 (see the boxes in Image B of Figure 6). These bubbles could affect the temporal response of the sensors. Although in this particular experiment, their presence did not significantly affect the measurement accuracy, it is noted that air bubbles near the sensors can lead to incorrect pH measurements because the optodes require sufficient contact with the measured fluid in order to obtain an accurate readout.

### 3.2. FOP-MIV Sensors

#### 3.2.1. Calibration 

**Figure 7:**
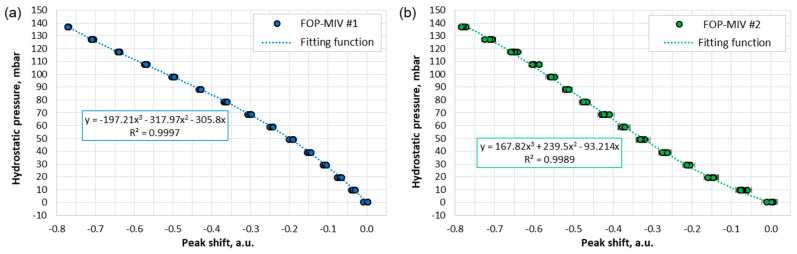
Calibration results and the 3rd order polynomial fits for: (**a**) FOP-MIV #1 and (**b**) FOP-MIV #2.

The FOP-MIV sensors were embedded into custom 3D-printed plugs, as shown in Figure 3e, and then were calibrated against hydrostatic pressure, as described in Section 2.5. Figure 7 shows the calibration results for both sensors. The hydrostatic pressure was calculated using Equation (1), whereas the peak shift was determined based on the data acquired by the interrogation system (described in Section 2.2.2 and Section 2.5). Since the hydrostatic pressure exerted on the sensor diaphragm reduces the length of the FP cavity, the characteristic peak for each sensor in the Fourier domain is shifted toward zero (see Figure 5a); hence, the peak shifts have negative values.

The relationship between the external pressure exerted on a circular diaphragm (*P_ext_*) and the deflection of the diaphragm (*d*_0_) in its center, where it is the greatest, can be described as follows [37] (p. 38):

(2) $$P_{ext}\left(d_{0}\right)=\left(\frac{16}{3}\cdot \frac{h^{3}\cdot E}{R^{4}\cdot \left(1-\mu^{2}\right)}+4\cdot \frac{h\cdot \sigma_{0}}{R^{2}}\right)\cdot d_{0}+\left(\frac{256}{105}\cdot \frac{h\cdot E}{R^{4}\cdot \left(1-\mu^{2}\right)}\right)\cdot {d_{0}}^{3}$$

where *µ*, *E*, *R*, and *h* are respectively the Poisson’s ratio, Young’s modulus, diaphragm radius, and thickness of the diaphragm, while *σ*_0_ is residual stress. The first parentheses contain two terms that describe the contribution of the bending moments and residual stress, whereas the term in the second parentheses describes stress due to straining of the diaphragm [37]. Here, it is noted that the bending moments and residual stress show a linear interrelationship between *P_ext_* and *d*_0_, while the stress due to straining contributes with its third power. When the terms in the first parenthesis of Equation (2) are significantly larger than the term in the second parenthesis, then Equation (2) can be written as:

(3) $$d_{0}=\left(\frac{3}{16}\cdot \frac{R^{4}\cdot \left(1-\mu^{2}\right)}{h^{3}\cdot E}+\frac{R^{2}}{4\cdot h\cdot \sigma_{0}}\right)\cdot P_{ext}.$$

This linear equation is usually appropriate to calculate the deflection of a circular diaphragm (*d*_0_) when the defection is less than 30% of the diaphragm thickness (*h*). 

The calibration data in Figure 7 do not follow a linear trend; therefore, a 3^rd^ order polynomial fit with the intercept at (0, 0) was used to interpolate the results. These fitting functions (their equations provided in Figure 7) accurately describe the relationship between the shift of the characteristic peaks and the hydrostatic pressure exerted on the sensors (up to 140 mbar). It is noted that the coefficients of the polynomial equations are different for each sensor, which suggests that there are some differences in the diaphragm parameters, e.g., in residual stress that may result from some production variation. 

All the data points in Figure 7 have error bars, although some are not visible because they are relatively small. The vertical error bars correspond to the sensor positioning accuracy in the glass cylinder below the water level, which was taken to be ±1 cm. In turn, the horizontal error bars correspond to the standard deviations (σ) for at least 120 values of the peak shifts acquired for a given hydrostatic pressure. The horizontal error bars for FOP-MIV #1 (see Figure 7a) are not visible because the σ values for this sensor are very small (<0.004), which corresponds to a pressure variation of less than ±0.6 mbar (valid for the entire calibration range). Such small variations result from the fact that this sensor gives a relatively narrow signal in the Fourier domain (see the bottom-left image in Figure 5a), so the peak location is well defined. In turn, the horizontal error bars for FOP-MIV #2 are visible (see Figure 7b) because the reflection spectrum of this sensor cavity has less regular “fringes” and therefore produces a wider signal in the Fourier domain (see the bottom-right image in Figure 5a). These irregularities probably result from unwanted reflections created by a parasitic cavity elsewhere in the system with an effective length similar to the length of the sensor cavity. Such a cavity could be formed in one of the mating sleeves (see Figure 2b). The σ values for FOP-MIV #2 were less than 0.015, and this corresponds to a pressure variation of less than ±2.5 mbar.

#### 3.2.2. Proof-of-Concept Experiments (Injection of Water)

**Figure 8:**
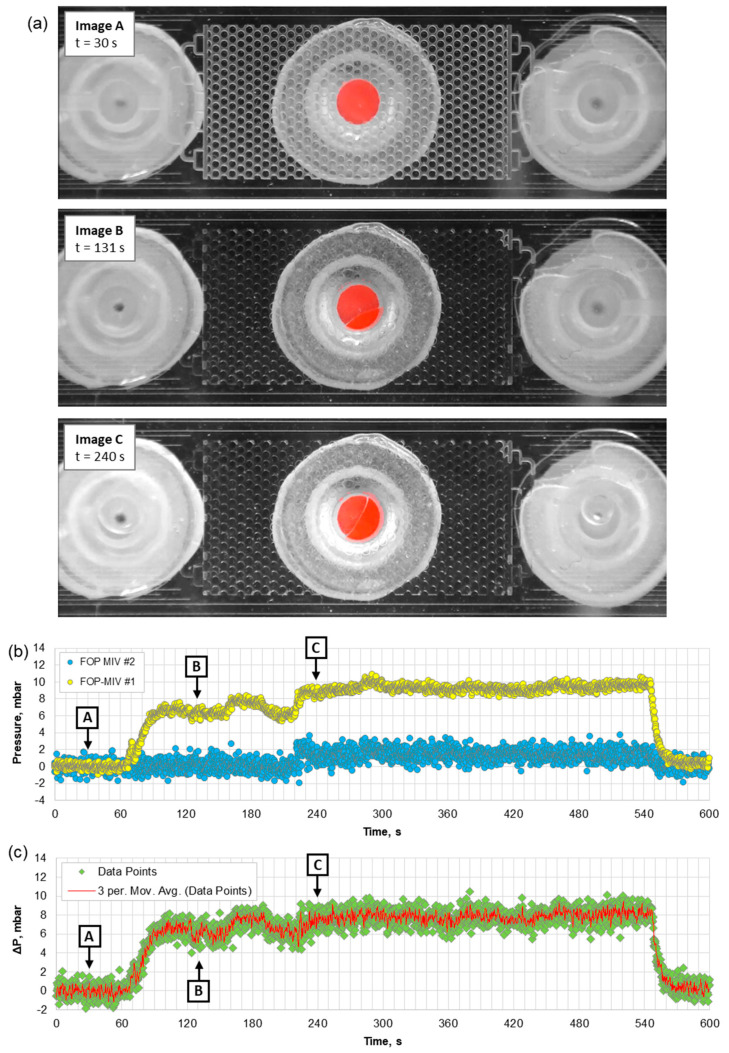
(**a**) Snapshots taken during the injection of water into the microfluidic pattern filled with air with a rate of 4 µL/min, (**b**) pressures measured at Ports 1 and 2, and (**c**) changes in the pressure difference (ΔP). The A, B, and C in (**b**,**c**) indicate the moments when the snapshots were taken.

Initially, the microfluidic pattern was filled with air, so in the first experiment, it was possible to observe simultaneously: (a) how the air in the pattern is displaced by water (images were captured by the camera) and (b) how the pressures in Ports 1 and 2 change during this event. Figure 8 shows the results of this experiment. 

The DI water was injected into the microfluidic pattern with a rate of 4 µL/min to displace the air. Initially (see Figure 8a, Image A), the FOP-MIV sensors did not detect any change in pressure because water had not yet reached Port 1, whilst the pressure increase due to flow of air at 4 µL/min was apparently below the sensor sensitivity. An increase of pressure was detected by FOP-MIV #1 when water arrived at Port 1 and started to flow through it. When water arrived at Port 2 (see Figure 8a, Image B), FOP-MIV #2 sensor did not immediately detect an increase of pressure. This was delayed by approximately 90 s until water reached and started to form a droplet at the outlet (see t = 220 s in Figure 8b). After an additional 90 s, the pressures in Ports 1 and 2 obtained equilibrium (P_1_ = 9.3 ± 0.4 mbar and P_2_ = 1.4 ± 0.7 mbar), and the pressure difference (ΔP) was measured to be 7.9 ± 0.8 mbar. The distribution of DI water within the pattern (see Figure 8a, Image C) did not change for the rest of the injection. 

Image C in Figure 8a shows that DI water injected with a rate of 4 µL/min did not completely saturate the microfluidic pattern, leaving air trapped in some areas. Small air bubbles were formed in all three ports, in a few outlet channels, and at the periphery of the pattern. Since air bubbles can make it difficult to correctly measure the absolute permeability of the pattern, they should be extracted from the microfluidic pattern using a vacuum pump or should be considered when calculating this parameter. 

The volume of the pattern confined by Ports 1 and 2 was estimated to be 3.1 µL. However, the camera allowed us to observe that the time needed for water to flow from Port 1 to Port 2 at an injection rate of 4 µL/min was approximately 70 s, which corresponds to a volume of 4.7 µL. Most likely, the additional 1.6 µL results from the small circular recesses created in the 3D-printed connectors (see Figure 3e), which were designed to protect the sensors from mechanical damage, and the possibility that these connectors were not fully inserted into the ports, thereby leaving a small gap between the connector facet and the laser-machined surface. Here, it should be noted that commercially available male mini-Luer connectors also introduce extra volumes (approximately 8 µL) into the flow systems [38] (p. 68).

**Figure 9:**
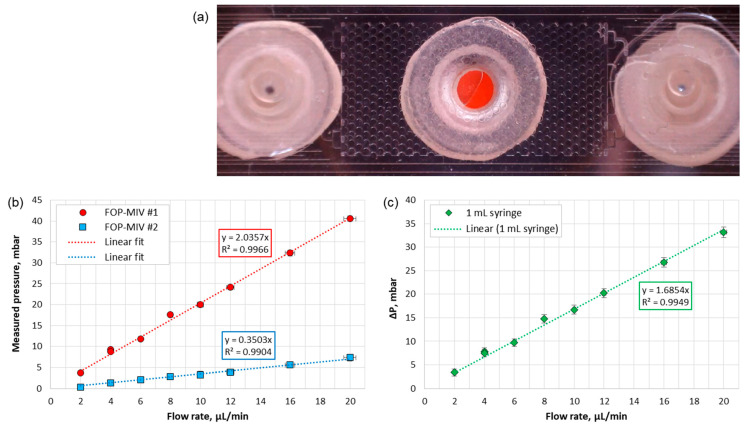
(**a**) Snapshot of the pattern taken when the flow rate of water was 20 µL/min, (**b**) pressures at Ports 1 and 2 and (**c**) pressure difference (ΔP) measured for different flow rates during the steady flow of water through the microfluidic pattern.

Following the displacement of air by water, the DI water was further injected into the pattern but with different rates. During this experiment, the distribution of water within the pattern did not change, as can be seen by comparing an image in Figure 9a with Image C in Figure 8a. The mean values of the pressures measured in Ports 1 and 2 for different flow rates during the steady flow of water through the pattern are shown in Figure 9b. Since water is an incompressible fluid, the absolute pressures as well as the pressure difference (see Figure 9c) increase linearly as the flow rate increases. Figure 9b,c also show linear fits to the experimental data that can be used to determine the permeability of the pattern.

## 4. Final Remarks and Conclusions

In this paper, we described an easy-to-implement method that enables flexible and “reversible” integration of commercially available fiber optic sensors within glass microfluidic devices. To demonstrate this, we designed custom connectors that were manufactured from UV-curable resin using a standard stereolithography 3D printer. To evaluate the performance of the connectors, they were used to attach “off-the-shelf” fiber optic sensors to the inlet and outlet channels of a microfluidic pattern. In this way, it was possible to perform in situ monitoring of pH and pressure during the displacement and flow of fluids. The experimental results have shown that the designed connectors ensure robust and watertight connection, allowing the sensors to be easily connected and safely disconnected if necessary, without damaging the microfluidic device. Although the connectors were used to integrate fiber optic sensors with a glass microfluidic device, they can also be used to attach such sensors to any microfluidic device made of a rigid material. This includes, for example, polymethylmethacrylate (PMMA), polycarbonate (PC), cyclic olefin copolymer (COC), and polylactic acid (PLA).

The use of the bespoke 3D-printed connectors has several advantages when integrating fiber optic sensors within microfluidic devices. Firstly, the connectors enable straightforward sensor replacement if necessary (e.g., due to component failure). Secondly, they allow one microfluidic device to be used in multiple experiments because the sensors can be easily disconnected when the microfluidic pattern must be cleaned or sterilized. This reduces overall experiment costs, because the device and sensors can be reused multiple times, whilst also providing experimental flexibility. Finally, it is possible to use different sensors with the same microfluidic device. In this paper, we demonstrated that the pH SensorPlugs and the FOP-MIV pressure sensors can be used with one microfluidic device. However, in the same way, it would be possible to attach other commercially available sensors, for example, O_2_ or CO_2_ SensorPlugs manufactured by PreSens Precision Sensing GmbH (Germany) that enable real-time measurement of oxygen and carbon dioxide, respectively. Since these sensors have the same physical footprint as the pH SensorPlugs, they could be easily incorporated with the microfluidic device without making any modification to the connectors.

The experiments performed with the sensors have shown that the 3D-printed connectors may require further improvements. The main concern is the potential for the formation of air bubbles near the sensors, which may affect the measurement accuracy as many (mainly chemical) sensors require sufficient contact between the measured fluid and the sensing element. One solution may be to reduce the size of the circular recess in the 3D-printed plugs (see Figure 3e). Another improvement would be miniaturization of the connectors, so they can allow more sensors to be integrated within a microfluidic device.

## Figures and Tables

**Table 1:** Specification of pH SensorPlugs and FOP-MIV sensors. Data extracted from [31,32].

Parameter	pH SensorPlug	FOP-MIV
Sensor diameter	≈1.8 mm ^1^	550 µm
Measurement range	pH 5.5–8.5	±0.4 bar ^4^
Sensor accuracy	±0.05 pH ^2^	±1.3 mbar ^5^
Time response	<120 s ^3^	-
Sampling rate	≤0.5 Hz	Up to 250 Hz ^6^
Sensitivity thermal effect	-	<0.4 mbar/°C
Operational temperature	0 to 50 °C	15 to 45 °C

^1^ Outer diameter of the mini-Luer plug is approximately 2.8 mm. ^2^ After single-spot calibration at pH = 7. ^3^ Equilibrated sensor kept in well-stirred solution at 37 °C. ^4^ Relative to atmospheric pressure. ^5^ When connected to a FISO SKR reading unit, the system accuracy is ±4 mbar. ^6^ This is a system measurement rate when used with a FISO SKR reading unit; however, the sensor response is probably significantly faster than 4 ms based upon the physical dimensions.

## Data Availability

Datasets and videos supporting the results shown in Figure 6, Figure 7, Figure 8 and Figure 9 are available from Heriot-Watt’s Research Portal (https://researchportal.hw.ac.uk/) (accessed on 9 November 2021).
